# Diabetes and Change in Bone Mineral Density at the Hip, Calcaneus, Spine, and Radius in Older Women

**DOI:** 10.3389/fendo.2013.00062

**Published:** 2013-05-30

**Authors:** Ann V. Schwartz, Susan K. Ewing, Anne M. Porzig, Charles E. McCulloch, Helaine E. Resnick, Teresa A. Hillier, Kristine E. Ensrud, Dennis M. Black, Michael C. Nevitt, Steven R. Cummings, Deborah E. Sellmeyer

**Affiliations:** ^1^Department of Epidemiology and Biostatistics, University of California San Francisco, San Francisco, CA, USA; ^2^Endocrine Division, Department of Medicine, University of California San Francisco, San Francisco, CA, USA; ^3^Department of Geriatrics, University of Maryland School of Medicine, Baltimore, MD, USA; ^4^Kaiser Permanente Center for Health Research Northwest/Hawaii, Portland, OR, USA; ^5^VA Medical Center, University of Minnesota, Minneapolis, MN, USA; ^6^California Pacific Medical Center, San Francisco, CA, USA; ^7^Division of Endocrinology, Johns Hopkins School of Medicine, Baltimore, MD, USA

**Keywords:** type 2 diabetes mellitus, bone mineral density, women, older adults, longitudinal studies

## Abstract

Older women with type 2 diabetes mellitus (DM) have higher bone mineral density (BMD) but also have higher rates of fracture compared to those without DM. Limited evidence suggests that DM may also be associated with more rapid bone loss. To determine if bone loss rates differ by DM status in older women, we analyzed BMD data in the Study of Osteoporotic Fractures (SOF) between 1986 and 1998. SOF participants were women ≥65 years at baseline who were recruited from four regions in the U.S. DM was ascertained by self-report. BMD was measured with dual-energy x-ray absorptiometry (DXA) at baseline and at least one follow-up visit at the hip (*N* = 6624) and calcaneus (*N* = 6700) and, on a subset of women, at the spine (*N* = 396) and distal radius (*N* = 306). Annualized percent change in BMD was compared by DM status, using random effects models. Of 6,867 women with at least one follow-up DXA scan, 409 had DM at baseline. Mean age was 70.8 (SD 4.7) years. Baseline BMD was higher in women with DM at all measured sites. In models adjusted for age and clinic, women with prevalent DM lost bone more rapidly than those without DM at the femoral neck (−0.96 vs. −0.59%/year, *p* < 0.001), total hip (−0.98 vs. −0.70%/year, *p* < 0.001), calcaneus (−1.64 vs. −1.40%/year, *p* = 0.005), and spine (−0.33 vs. +0.33%/year, *p* = 0.033), but not at the distal radius (−0.97 vs. −0.90%/year, *p* = 0.91). These findings suggest that despite higher baseline BMD, older women with DM experience more rapid bone loss than those without DM at the hip, spine, and calcaneus, but not the radius. Higher rates of bone loss may partially explain higher fracture rates in older women with DM.

## Introduction

Type 2 diabetes mellitus (DM) and osteoporosis are two chronic conditions whose prevalence and associated costs continue to increase, particularly among the elderly. Internationally, over 10% of adults age 60 years and older have DM; in the U.S. the prevalence of DM in this age group is nearly 30% (Wild et al., 2004; Cowie et al., 2009). The annual number of hip fractures worldwide was estimated as 1.26 million in 1990, and is projected to approximately double by 2025 (Gullberg et al., 1997). In older adults, considerable overlap in DM and osteoporosis would be expected simply due to the high prevalence of each condition. In addition, DM is associated with increased risk of fracture (Janghorbani et al., 2007; Vestergaard, 2007). Paradoxically, cross-sectional studies have demonstrated that DM is associated with normal or higher bone mineral density (BMD) (Buysschaert et al., 1992; Bauer et al., 1993; Orwoll et al., 1996; Vestergaard, 2007). Thus, for any given BMD *T*-score, the fracture risk in those with DM tends to be higher than the corresponding risk for non-diabetic patients (Schwartz et al., 2011).

Although DM is associated with higher baseline BMD, there is some evidence that people with DM may have more rapid bone loss. This could partially account for higher fracture risk at a given BMD since rapid bone loss contributes to fracture risk independent of baseline BMD (Hillier et al., 2007; Ahmed et al., 2010; Cawthon et al., 2012). However, previous reports on the rate of bone loss in older adults with DM have been inconsistent. While several studies have reported accelerated bone loss at the hip in older women with DM, including in the Study of Osteoporotic Fractures (SOF) (Keegan et al., 2004; Cauley et al., 2009; Khalil et al., 2011), slower bone loss has also been reported at the spine (Khalil et al., 2011) and radius (Krakauer et al., 1995). Our goals in this study were to clarify the effects of diabetes on the rate of bone loss and to gain insight into the seemingly paradoxical and poorly understood relationships among diabetes, BMD, and fracture. To achieve these goals, we studied the associations between diabetes and rate of bone loss at several skeletal sites in older women enrolled in the Study of Osteoporotic Fractures (SOF), using longitudinal data from 1986 to 1998.

## Materials and Methods

### Participants

The Study of Osteoporotic Fractures (SOF) is a prospective cohort of 9,704 white women aged ≥65 years. Participants were recruited from the community in four U.S. regions: Portland, Oregon; Minneapolis, Minnesota; Baltimore County, Maryland; and the Mononghela Valley near Pittsburgh, Pennsylvania. Enrollment began in 1986, and the current analyses are based on follow-up data through 1998 (Cummings et al., 1990). Women were recruited irrespective of BMD and fracture history; those unable to walk without assistance and those with bilateral hip replacements were excluded. All women provided written consent, and SOF was approved by the Institutional Review Board at each site.

### Ascertainment of diabetes mellitus

At baseline, participants were asked if a physician had ever told them that they had diabetes or “sugar diabetes.” Women who answered “yes” to this question were identified as having prevalent DM. Twenty-five women did not answer this question and were excluded. Using the same survey question, incident DM was defined at years 3.5, 6, 8, and 10 (corresponding to SOF clinic visits 3, 4, 5, and 6) and via medication inventory at years 6, 8, and 10. Women who did not report diabetes but who were taking diabetic medications were classified as having DM. Nine women included in these analyses reported thiazolidinedione (TZD) use at year 10.

### Measurement of bone mineral density

#### Calcaneal BMD

**Figure 1:**
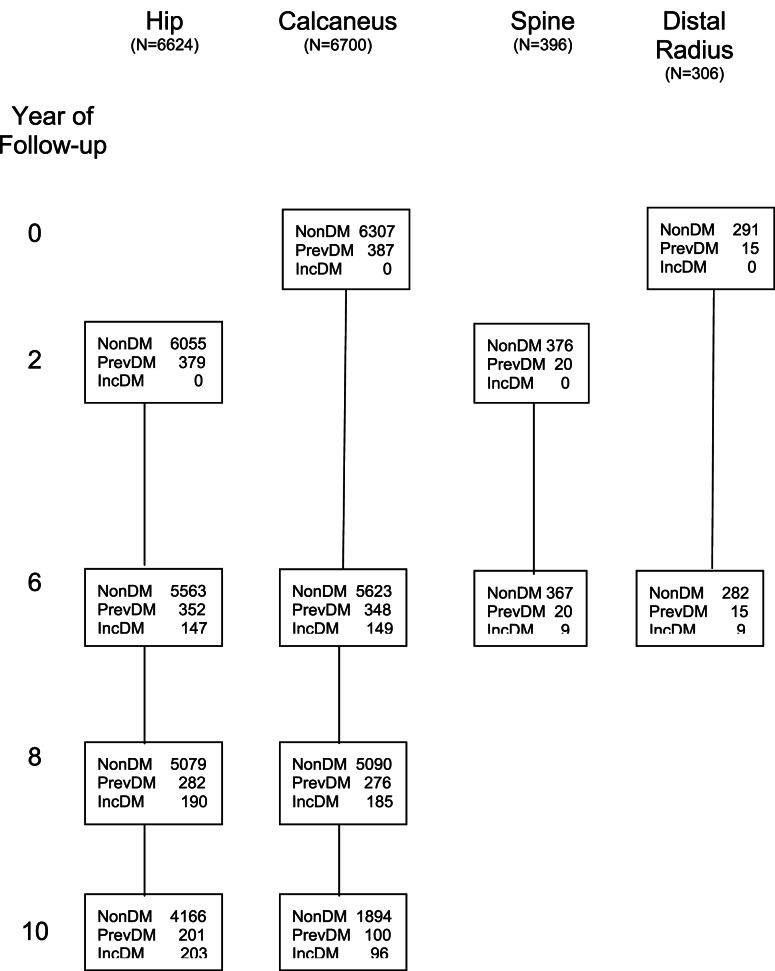
**Number of participants in each bone loss analysis by diabetes status at each visit**. *N*, number of participants included in analysis of each skeletal site; non-DM, no DM at current or previous visits; PrevDM, DM at baseline; IncDM, new diagnosis of DM between baseline and current visit.

Peripheral BMD was measured at the calcaneus using single photon absorptiometry (Osteoanalyzer; Dove Medical Systems) (Cummings et al., 1990) at the baseline, year 6 and year 8 visits in all women and at the year 10 visit in a subset of participants (Figure 1). Of the 9,679 women with baseline DM data, 6,700 women had ≥2 calcaneal measurements and were included in analyses examining the association between DM and change in calcaneal BMD.

#### Radial BMD

Distal and proximal radial BMD were measured by single photon absorptiometry. The distal measurement site was just proximal to the junction of the ulna and radius, and the proximal site was 25% of the total ulnar length distant from the distal site (Cummings et al., 1990). Radial scans were obtained in all women at baseline and in a small subset of women at year 6. Of the 9,679 women with baseline DM data, 306 had distal BMD measurements and 290 had proximal BMD measurements at both visits and were included in analyses examining the association between DM and changes in distal and proximal radial BMD (Figure 1).

#### Hip and spine BMD

Bone mineral density of the total hip, femoral neck, and total lumbar spine was first measured at year 2 (visit 2) using dual-energy x-ray absorptiometry (DXA) with Hologic QDR-1000 scanners (Hologic, Inc., Bedford, MA, USA). Hip BMD was measured again at years 6 and 8 on the same scanners. At year 10, hip BMD was measured with QDR-1000 scanners for 4,224 women and with QDR-2000 scanners for 346 women. Of the 9,679 women with baseline DM data, 6,624 had ≥2 hip BMD measurements and were included in analyses examining the association between DM and BMD at the total hip and femoral neck (Figure 1). A subset of the 9,679 women had spine BMD measured at year 6 (*N* = 479), with 396 having spine BMD measurements at both years 2 and 6; these women were included in analyses examining the association between DM and spine BMD.

### Other measurements

Weight was measured on a standard balance beam scale, and weight change was calculated by subtracting baseline weight (or year 2 weight for the spine and hip analyses) from current weight. Height was measured by a Harpenden stadiometer (Holtain Ltd., Dyved, UK). Self-reported height at age 25 was collected at baseline; height change was calculated by subtracting height at age 25 from baseline height. Self-reported age at the last menstrual period (LMP) was collected at baseline, and number of years since menopause was calculated by subtracting age at LMP from baseline age (or from year 2 age for hip and spine BMD models). Current use of vitamin D and calcium supplements, estrogen preparations, thiazide diuretics, and oral steroids was self-reported at baseline and year 2. Beginning in year 6, participants were asked to bring all prescription and non-prescription medications to the clinic visit for a medication inventory. Self-reported use of calcitonin injections and fluoride pills started at year 2, and self-reported use of etidronate started at year 6. Tobacco use and walking for exercise were self-reported at each visit. Various aspects of physical performance were assessed by trained examiners at each visit. These included grip strength (measured with a handheld Jamar dynamometer using the average of two trials per hand) (Cummings et al., 1995; Seeley et al., 1995), gait speed (measured on a 6-m walking course using the time to complete two trials (Cummings et al., 1995), and ability to rise from a chair five times without using arms (Cummings et al., 1995; Seeley et al., 1995). Peripheral nerve function was assessed at year 2 using esthesiometer testing on the warmed great toe of both feet, using six filaments of increasing size (3.22–6.10, logarithm of force applied, in 0.1 g). Women who felt only the 6.10 filament or no filament on either toe were identified as having poor light touch discrimination.

### Statistical analyses

Characteristics of participants were examined according to baseline DM status, using *t*-tests for continuous variables and chi-square tests for categorical variables. Since spine and radial BMD were measured at only two time points, linear regression was used to examine the association between DM and change in BMD at these sites, with results presented as least square means. By contrast, since hip and calcaneal BMD were measured at several time points, random effects models were used to examine the association between DM and change in BMD at these sites. These models account for the between-subject variation and within-subject correlations among repeated BMD measurements. Time was modeled as a continuous covariate, measured as the number of years between the first BMD and the follow-up BMD scans for each site. Random effects models included the intercept and slope of the BMD measurements over time, thereby allowing for individual time trends for each participant.

All models were adjusted for age and clinic site. The following covariates were initially considered for inclusion in the multivariate models: baseline (or year 2) weight, weight change, baseline (or year 2) height, height loss since age 25, and current use of any of the following: vitamin D, calcium supplements, estrogen, osteoporosis medications (alendronate, raloxifene, tamoxifen, etidronate, fluoride pills, or calcitonin injections), thiazide, and oral steroids. Also considered for inclusion in the models were current grip strength, walking speed, ability to rise from chair, walking for exercise, years since menopause, current tobacco use, and poor light touch discrimination at year 2. These covariates were included in the multivariate model if they were significantly associated both with DM in univariate analyses and with change in BMD in the age-clinic-adjusted models at *p*-value < 0.10. Separate multivariate models were constructed for each skeletal site. Weight loss was added separately to the multivariate models to assess its role as a potential intermediary between DM and change in BMD because it is known to predict bone loss from other studies (Ensrud et al., 2003), and DM is associated with weight loss in the SOF cohort. Change in BMD is reported as annualized percent change. For hip and calcaneal BMD, the mixed model estimates were used to estimate BMD at each year of follow-up and plotted to visualize changes in BMD over time for each of the DM groups. All analyses were conducted using SAS version 9.1 (SAS Institute Inc., Cary, NC, USA).

## Results

Of the 9,679 women at baseline with known diabetes status, 6,867 had ≥2 BMD measurements during the first 10 years of follow-up. Of these, 409 (6%) self-reported a physician diagnosis of diabetes at baseline and were categorized as having prevalent DM. Of the remaining 6,458 women who were not diabetic at baseline, 399 (6%) developed incident DM during follow-up. Characteristics of the 6,867 women included in one or more of these analyses are presented in Table 1. Compared to non-diabetic women, women with prevalent DM had higher baseline BMD at all six sites. Women with prevalent DM had lower grip strength, slower walking speed, and were less likely to walk for exercise and to report estrogen use. Use of alendronate or raloxifene was similar in women with and without DM.

**Table 1:** **Characteristics^a^ of women by diabetes status**.

	Non-DM	Prevalent DM	*p*-Value
	(*N* = 6458)	(*N* = 409)	
Age (years)	70.7 ± 4.7	71.0 ± 4.6	0.35
Weight (kg)	67.1 ± 12.0	72.6 ± 14.8	<0.001
Change in weight (V6-BL) (%/year)	−0.25 ± 0.96	−0.41 ± 0.99	0.012
Height (cm)	159.6 ± 5.9	158.9 ± 6.1	0.017
Height loss since age 25 (cm)	3.1 ± 2.8	2.9 ± 2.6	0.095
Years since menopause (years)	23.6 ± 7.8	24.7 ± 8.1	0.006
Current smoker	573 (8.9)	34 (8.3)	0.69
Current vitamin D use	2958 (46.6)	149 (36.9)	<0.001
Current calcium use	2871 (44.6)	134 (32.8)	<0.001
Current estrogen use	1226 (19.5)	42 (10.4)	<0.001
Alendronate taken in last 2 years (V6)	479 (8.9)	18 (6.6)	0.18
Current raloxifene use (V6)	8 (0.2)	1 (0.4)	0.36
Current thiazide use	1513 (23.7)	153 (37.8)	<0.001
Current statin use (V4)	248 (3.9)	22 (5.5)	0.12
Current oral steroid use	110 (1.7)	4 (1.0)	0.27
Grip strength (kg)	21.3 ± 4.1	20.8 ± 4.4	0.020
Walking speed (m/s)	1.05 ± 0.20	0.96 ± 0.21	<0.001
Inability to rise from chair	142 (2.2)	14 (3.4)	0.10
Walk for exercise	3478 (53.9)	189 (46.2)	0.003
Poor light touch discrimination (V2)	240 (4.3)	35 (9.9)	<0.001
Baseline BMD
Calcaneus BMD (g/cm^2^)	0.41 ± 0.09	0.44 ± 0.10	<0.001
Distal radius BMD (g/cm^2^)	0.36 ± 0.08	0.39 ± 0.08	<0.001
Proximal radius BMD (g/cm^2^)	0.64 ± 0.1	0.67 ± 0.1	<0.001
Femoral neck BMD (V2) (g/cm^2^)	0.65 ± 0.11	0.69 ± 0.12	<0.001
Total hip BMD (V2) (g/cm^2^)	0.76 ± 0.13	0.81 ± 0.14	<0.001
Total lumbar spine BMD (V2) (g/cm^2^)	0.86 ± 0.17	0.9 ± 0.17	<0.001

Mean (SD), or *N* (%). BMD, bone mineral density; DM, diabetes mellitus.

^a^Measurement at baseline visit unless otherwise indicated.

### Diabetes and change in hip BMD

**Figure 2:**
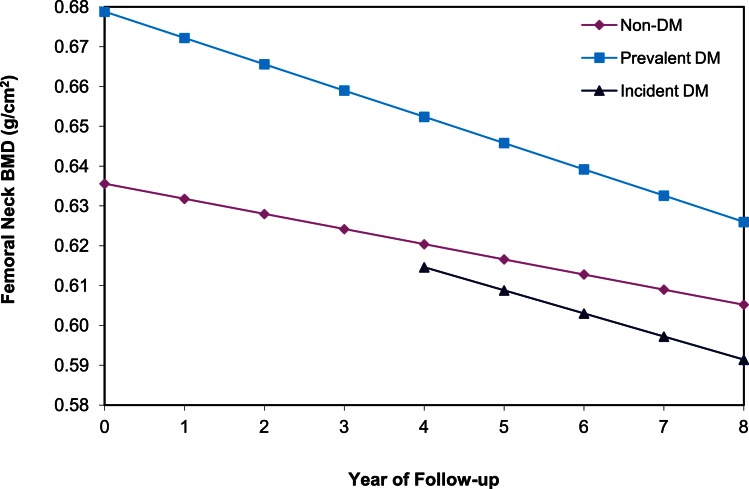
**BMD over time at the femoral neck among older women by diabetes status**. Mixed model estimates, adjusted for age and clinic site, were used to estimate BMD at each year of follow-up.

Among the 6,624 women with ≥2 hip BMD measurements between years 2 and 10, 391 and 303 had prevalent and incident DM, respectively. In age and clinic-adjusted models for the femoral neck, women with prevalent DM, incident DM, and those without DM lost an average of 0.96%, 0.90, and 0.59 BMD %/year, respectively (Table 2). At the total hip, both prevalent and incident DM lost 0.98%/year, while non-DM women lost an average of 0.70%/year. Although bone loss was more rapid in women with prevalent DM, average BMD remained higher compared with non-DM women throughout 8 years of follow-up (Figure 2).

**Table 2:** **Adjusted mean BMD change at the hip and calcaneus by diabetes status**.

Site of BMD Change	Prevalent DM			Incident DM			Non-DM	
	
	
	
	Change (%/year)	95% CI	p*	Change (%/year)	95% CI	p**	Change (%/year)	95% CI
**FEMORAL NECK**								
Adjusted for age and site	−0.96	−1.17, −0.77	<0.001	−0.90	−1.22, −0.59	0.009	−0.59	−0.66, −0.53
MV without weight change^a^	−0.86	−1.10, −0.63	<0.001	−0.79	−1.14, −0.44	0.06	−0.54	−0.62, −0.46
MV with weight change^a^	−0.79	−1.03, −0.57	<0.001	−0.69	−1.04, −0.36	0.18	−0.51	−0.59, −0.44
**TOTAL HIP**								
Adjusted for age and site	−0.98	−1.15, −0.82	<0.001	−0.98	−1.22, −0.75	0.001	−0.70	−0.76, −0.65
MV without weight change^a^	−0.86	−1.06, −0.68	<0.001	−0.77	−1.03, −0.52	0.06	−0.59	−0.66, −0.53
MV with weight change	−0.75	−0.92, −0.58	<0.001	−0.61	−0.86, −0.38	0.51	−0.55	−0.61, −0.50
**CALCANEUS**								
Adjusted for age and site	−1.64	−2.06, −1.25	0.005	−1.43	−2.64, −0.40	0.94	−1.40	−1.54, −1.27
MV without weight change^b^	−1.29	−1.75, −0.86	0.06	−0.83	−2.13, 0.27	0.57	−1.08	−1.24, −0.93
MV with weight change	−1.19	−1.65, −0.76	0.14	−0.68	−1.96, 0.40	0.44	−1.04	−1.19, −0.88

*p-Value for differential rate of BMD loss for prevalent DM vs. non-DM.

**p-Value for differential rate of BMD loss for incident DM vs. non-DM.

^a^Adjusted for baseline age, clinic site, baseline height, baseline weight, height change since 25 years since menopause, current vitamin D use, current calcium use, current estrogen use, current use of osteoporosis medications, current thiazide diuretic use, current grip strength, current walking speed, current inability to rise from chair without use of arms, and decreased light touch.

^b^Adjusted for baseline age, clinic site, baseline weight, baseline height, height change since age 25, years since menopause, current vitamin D use, current estrogen use, current thiazide use, current grip strength, current walking speed, current inability to rise from chair without use of arms, and current walking for exercise.

In multivariate models, mean BMD loss remained significantly greater for the women with prevalent DM compared to women without DM at both the femoral neck (−0.86 vs. −0.54%/year, *p* < 0.001) and total hip (−0.86 vs. −0.59%/year, *p* < 0.001). Additional adjustment for concurrent weight change slightly attenuated, but did not eliminate, the association between prevalent DM and accelerated BMD loss (Table 2). For incident DM compared to women without DM, multivariate adjustment attenuated the associations for femoral neck (−0.79 vs. −0.54%/year, *p* = 0.06) and total hip (−0.77 vs. −0.59%, *p* = 0.06) BMD, and they were no longer statistically significant (Table 2). Further adjustment for concurrent weight change resulted in additional attenuation of these relationships comparing women with incident DM to those without DM.

### Diabetes and change in calcaneal BMD

**Figure 3:**
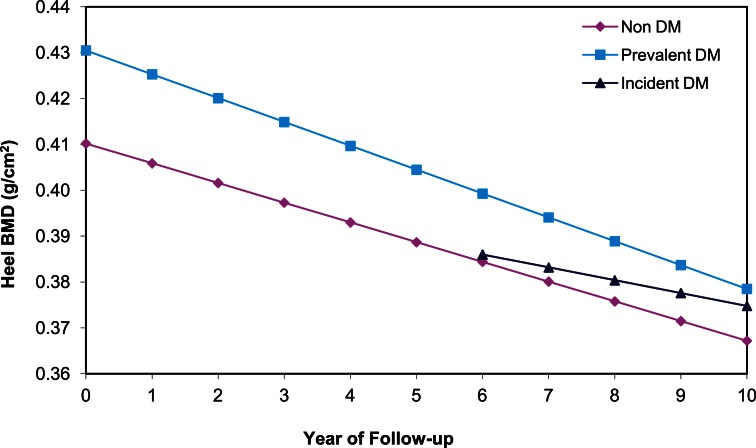
**BMD over time at the calcaneus among older women by diabetes status**. Mixed model estimates, adjusted for age and clinic site, were used to estimate BMD at each year of follow-up.

Of the 6,700 women with ≥2 calcaneal BMD measurements between baseline and year 10, 387 and 306 had prevalent and incident DM, respectively. Adjusted for age and clinic, women with prevalent DM lost an average of 1.6%/year, while those with incident DM and non-DM women lost 1.4%/year (Table 2). Calcaneal BMD was highest among women with prevalent DM, but declined more rapidly over time. Although bone loss was more rapid in those with prevalent DM, average BMD remained higher compared with non-DM women even after 10 years of follow-up (Figure 3). The difference in mean loss between the prevalent DM and non-DM groups was significant in the age- and clinic-adjusted model, of borderline significance in the multivariate model, and not significant in a model adjusting for weight change. There was no difference in BMD loss between women with incident DM and those without DM in any of the calcaneal models.

### Diabetes and change in lumbar spine BMD

Of the 396 women with lumbar spine BMD measurements at years 2 and 6, 20 had prevalent DM and 9 developed incident DM. Adjusted for age and clinic, women with prevalent DM lost BMD (−0.33%/year), while those without DM gained bone (0.33%/year; *p* = 0.03) (Table 3). Numbers were too small to assess incident DM separately. Results were similar with multivariable adjustment, including adjustment for concurrent weight change.

**Table 3:** **Adjusted mean BMD change at the spine and radius by diabetes status**.

Site of BMD Change	Prevalent DM			Non-DM	
	
	
	Change (%/year)	95% CI	*p*-value*	Change (%/year)	95% CI
**SPINE**					
Adjusted for age and site	−0.33	−0.92, 0.26	0.03	0.33	0.20, 0.47
MV without weight change^a^	−0.41	−0.98, 0.17	0.01	0.35	0.21, 0.48
MV with weight change	−0.36	−0.92, 0.21	0.02	0.34	0.21, 0.47
**DISTAL RADIUS**					
Adjusted for age and site	−0.97	−2.01, 0.08	0.91	−0.90	−1.14, −0.66
MV without weight change^b^	−1.12	−2.18, −0.07	0.68	−0.89	−1.14, −0.65
MV with weight change	−1.13	−2.18, −0.08	0.67	−0.89	−1.13, −0.65
**PROXIMAL RADIUS**					
Adjusted for age and site	0.74	−0.63, 2.11	0.14	−0.33	−0.65, −0.01
MV without weight change^b^	0.71	−0.67, 2.09	0.15	−0.35	−0.67, −0.02
MV with weight change	0.71	−0.68, 2.09	0.15	−0.35	−0.67, −0.02

*p-Value compared to non-DM.

^a^Adjusted for age, clinic site, weight, thiazide diuretic use, and vitamin D use.

^b^Adjusted for age, clinic site, weight, walking for exercise, height change since age 25, and vitamin D use.

### Diabetes and change in radial BMD

Of the 306 women with distal BMD measurements at baseline and year 6, 15 had prevalent DM at baseline and 9 women developed incident DM between baseline and year 6. After adjustment for age and clinic site, women with prevalent DM lost an average of 0.97%/year at the distal radius while non-DM women lost 0.90%/year (*p* = 0.91) (Table 3). Numbers were too small to assess incident DM separately. Multivariable adjustment did not substantially alter these results. Of the 290 women with two proximal BMD measurements, 15 had prevalent DM and 7 developed incident DM. Adjusted for age and clinic site, women with prevalent DM gained an average of 0.74%/year at the proximal radius while non-DM women lost 0.33%/year (*p* = 0.14). Multivariate adjustment did not substantially alter these results.

## Discussion

Despite their higher baseline BMD, older women with prevalent DM had more rapid bone loss at the total hip, femoral neck, lumbar spine, and calcaneus, but not at the distal or proximal radius, than their non-diabetic counterparts. These results strengthen the evidence for an association between DM and accelerated bone loss, but also clarify that this relationship is site-specific. Our findings extend a previous report from the SOF cohort that DM is associated with accelerated bone loss at the total hip (Cauley et al., 2009), and are in agreement with previous observations of more rapid bone loss at the hip among postmenopausal women with DM in the Health, Aging, and Body Composition Study (Health ABC) and in the placebo group of the Fracture Intervention Trial (FIT) (Keegan et al., 2004; Schwartz et al., 2005). In FIT, there was also a trend toward a faster rate of bone loss at the spine among diabetic women but the difference was not statistically significant (Keegan et al., 2004). Similarly, the Study of Women’s Health Across the Nation (SWAN) found an increased rate of bone loss at the total hip among women with DM in the post menopausal but not perimenopausal time period (Khalil et al., 2011). However, SWAN, in contrast to our findings, reported a slower rate of bone loss at the spine in women with DM. Another study found a slower rate of bone loss at the radius over 12 years in 19 adults with DM (average age 52 years), based on BMD *z*-scores (Krakauer et al., 1995). By comparison, we found no differences in the rate of bone loss at the radius.

Thiazolidinedione use may contribute to more rapid bone loss in those with DM. In randomized controlled trials, TZDs have been shown to increase bone loss at the spine and total hip (Berberoglu et al., 2007; Grey, 2008; Borges et al., 2011). However, our findings are not explained by TZD use. The vast majority of follow-up of the SOF cohort took place before the introduction of troglitazone in 1997 and of rosiglitazone and pioglitazone in 1999. Indeed, only nine participants in these analyses reported TZD use at year 10.

Our findings indicate that more rapid bone loss associated with DM is a feature of the hip, spine, and calcaneal sites, but not the radius. In comparison with the other three sites, the radius has a higher proportion of cortical bone and is a non-weight bearing site. In theory, the lack of effect of DM at the radius could result from a stronger association with loss of trabecular rather than cortical bone, but cross-sectional studies suggest the opposite (Melton et al., 2008; Petit et al., 2010; Shu et al., 2012; Patsch et al., 2013). To our knowledge, longitudinal studies using quantitative computed tomography (QCT) are not currently available to help disentangle the effect of DM on cortical compared with trabecular bone loss. Another possible explanation is a stronger effect of DM on bone loss in the presence of loading. Other studies indicate that bone geometry, although not bone density, may be negatively affected by DM with a reduction in bone strength relative to load (Petit et al., 2010; Ishii et al., 2012). DM is associated with higher levels of sclerostin (Garcia-Martin et al., 2012), indicating an effect on osteocytes, and with reduced bone formation (Krakauer et al., 1995; Shu et al., 2012). DM may inhibit the ability of osteocytes and osteoblasts to respond adequately to load. Thus, at skeletal sites that experience loading, older women with DM may experience greater net loss of bone.

Our results showing higher BMD at baseline are consistent with meta-analyses reporting higher BMD associated with DM (Vestergaard, 2007; Ma et al., 2012). Yet, surprisingly, DM was also associated with more rapid bone loss at the hip, spine, and calcaneus. The rate of bone loss was accelerated among those with incident DM at the hip, but not the calcaneus. Thus, at least for the hip, our results suggest that even at diagnosis, DM has an unfavorable impact on BMD. We also found more rapid weight loss among the diabetic women, despite higher baseline weight. Previous studies have also reported more rapid weight loss with DM (Moritz et al., 1994; Looker et al., 2001; Schwartz et al., 2005). While weight loss is a hallmark of poorly controlled DM, more rapid weight loss was also observed in those with relatively good control (Looker et al., 2001). Weight loss is a strong risk factor for bone loss (Hannan et al., 2000), and more rapid bone loss among diabetic women appears to account in part for more rapid bone loss in our study. However, if weight loss were the only mechanism, we would expect our models that included concurrent weight loss to abolish the relationship between bone loss and DM. Instead, although adjustment for concurrent weight change attenuated the effect of accelerated BMD loss among women with prevalent DM, it did not abolish the effect. This indicates that more rapid weight loss does not account for the more rapid bone loss that we observed in women with DM.

Other possible mechanisms for accelerated bone loss with DM include lower levels of insulin-like growth factor 1, changes in calcium homeostasis, increased advanced glycation end products, and decreased blood flow to the lower extremities (Raskin et al., 1978; Vogt et al., 1997; Schwartz et al., 2009). Higher levels of inflammatory cytokines or oxidative stress in those with DM may also drive bone loss (Clowes et al., 2005; Cauley et al., 2007; Manolagas and Almeida, 2007). Reduced exercise in those with DM may increase bone loss (Greendale et al., 1995), but adjustment for physical activity in our models did not account for the observed association between DM and rate of bone loss. Thus, the mechanisms underlying the association between DM and accelerated bone loss merit further exploration.

It has been shown that BMD *T*-score and FRAX underestimate fracture risk in DM women (Schwartz et al., 2011; Giangregorio et al., 2012). Accelerated bone loss with DM may be a contributing factor as the rate of bone loss predicts fractures independent of baseline BMD (Nguyen et al., 2005; Sornay-Rendu et al., 2005; Bruyere et al., 2007; Hillier et al., 2007; Berger et al., 2009; Ahmed et al., 2010; Cawthon et al., 2012). The reasons for the association between more rapid bone loss and fracture remain controversial. More rapid bone loss may simply be a marker for a lower BMD closer to the time of the fracture. In support of this hypothesis, in the Tromso study the rate of bone loss was no longer associated with fracture risk when models were adjusted for the final BMD measurement, closer to the time of fracture (Ahmed et al., 2010). In contrast, in the Study of Osteoporosis in Men (MrOS) cohort the rate of bone loss predicted hip fracture independent of baseline BMD and final BMD measurements (Cawthon et al., 2012), suggesting that more rapid bone loss may be a marker for changes in bone strength that cannot be fully captured by DXA. Fracture in diabetic women is associated with increased cortical porosity, a feature of bone that is not appreciated with DXA scans (Patsch et al., 2013). In our analyses, although DM women had more rapid bone loss, they continued to have higher average hip BMD compared with non-DM women, even after 8 years of follow-up.

Strengths of our study include up to 10 years of follow-up for BMD changes and the ability to adjust for multiple potential confounders, including use of estrogen and osteoporosis medications. A limitation of our study is the relatively small number of women with BMD scans of the spine and radius. Another limitation of this study is the lack of blood tests for ascertainment of DM. It is likely that some participants had undiagnosed diabetes and were incorrectly classified as not having diabetes (Schneider et al., 2012). This misclassification should not have differed by levels of the outcome, changes in BMD. Thus, any misclassification would tend to bias our measures of association between diabetes and change in BMD toward the null. In addition, SOF participants were community dwelling white women, and these results may not apply to other populations. Although analyses were adjusted for multiple factors, the possibility of residual confounding due to factors that were not measured or were measured with error cannot be eliminated.

In conclusion, older women with DM had accelerated bone loss at the hip, spine, and calcaneus, but not at the radius, compared to women without diabetes. Greater concurrent weight loss in the women with DM accounted for some, but not all, of the association between DM and accelerated BMD loss, suggesting that other diabetes-related mechanisms increase bone loss. Despite higher baseline BMD, accelerated bone loss may account, at least in part, for the increased fracture rate observed with DM.

## Conflict of Interest Statement

The authors declare that the research was conducted in the absence of any commercial or financial relationships that could be construed as a potential conflict of interest.
